# Oral anticoagulant treatment after bioprosthetic valvular intervention or valvuloplasty in patients with atrial fibrillation—A SWEDEHEART study

**DOI:** 10.1371/journal.pone.0262580

**Published:** 2022-01-13

**Authors:** Christina Christersson, Claes Held, Angelo Modica, Johan Westerbergh, Gorav Batra

**Affiliations:** 1 Department of Medical Sciences, Cardiology, Uppsala University, Uppsala, Sweden; 2 Uppsala Clinical Research Center, Department of Medical Sciences Cardiology, Uppsala University, Uppsala, Sweden; 3 Pfizer AB, Sollentuna, Sweden; 4 Uppsala Clinical Research Center, Uppsala University, Uppsala, Sweden; Karolinska Institutet, SWEDEN

## Abstract

**Aims:**

To describe the prevalence of atrial fibrillation (AF), use of oral anticoagulants (OAC) and change in antithrombotic treatment patterns during follow-up after valve intervention with a biological prosthesis or valvuloplasty.

**Methods and results:**

All patients with history of AF or new-onset AF discharged alive after valvular intervention (biological prosthesis or valvuloplasty) between 2010–2016 in Sweden were included (n = 7,362). Information about comorbidities was collected from national patient registers. Exposure to OAC was based on pharmacy dispensation data. In total 4,800 (65.2%) patients had a history of AF, and 2,562 (34.8%) patients developed new-onset AF, with 999 (39.0%) developing new-onset AF within 3 months after intervention. The proportion of patients with biological valve prosthesis was higher in patients with new-onset AF compared to history of AF (p<0.001). CHA_2_DS_2_-VASc score ≥2 was observed in 83.1% and 75.5% patients with history of AF and new-onset AF, respectively. Warfarin was more frequently dispensed than NOAC at discharge in patients with history of AF (43.9% vs 7.3%), and in patients with new-onset AF (36.6% vs 17.1%). Almost half of the AF population was not dispensed on any OAC at discharge (48.8% in patients with history of AF and 46.3% in patients with new-onset AF).

**Conclusion:**

In this real world study of patients with AF and recent valvular intervention, risk of new-onset AF after valvular intervention is high emphasizing need for frequent rhythm monitoring after intervention. A considerable undertreatment with OAC was observed despite being indicated for the majority of the patients. Warfarin was the OAC most frequently dispensed.

## Introduction

Non-vitamin K antagonist oral anticoagulants (NOAC) as primary prevention of stroke in patients with atrial fibrillation (AF) is recommended over warfarin, and is given high priority in guideline recommendations [1–3]. However, there are still some evidence gaps of the efficacy and safety of NOAC in patients with AF and valvular disease (VHD), especially in patients with a recently implanted valve prosthesis or after valvuloplasty [4–7]. AF in patients with concomitant VHD is important since the comorbidity is common and associated with worse clinical outcome [8]. In patients planned for open heart surgery, the occurrence of AF varies between 16 to 30%, with higher occurrence of AF in patients undergoing transcatheter aortic valve implantation (TAVI) [9–12].

In the large randomized NOAC trials in patients with AF, recent valve surgery was an exclusion criteria and only a minor proportion of patients with previous biological valve interventions was included [13–16]. In post-hoc analyses of patients with previous valvular surgery in the NOAC trials, no signals of worse outcome with NOAC versus warfarin have been described [17, 18]. Extensive evidence, from experimental and clinical studies, have shown that the coagulation system is triggered by artificial surfaces and the risk of valvular thrombosis is highest in the early phase after a surgical intervention [19, 20]. However, the knowledge of whether NOAC can be used in patients with AF early after surgical or transcatheter biological valve intervention is limited [4, 7]. Furthermore, it is not well described how these patients are managed in clinical practice in terms of oral anticoagulant (OAC) treatment. We therefore performed a real world study based on national registries in Sweden covering all biological valve interventions. The aims of the present study were to describe (1) demographics and clinical characteristics, (2) use of different OACs and (3) changes in antithrombotic treatment patterns during long-term follow-up in patients with history of AF versus new-onset AF after discharge from a valvular intervention.

## Material and methods

### Study population and data sources

**Fig 1:**
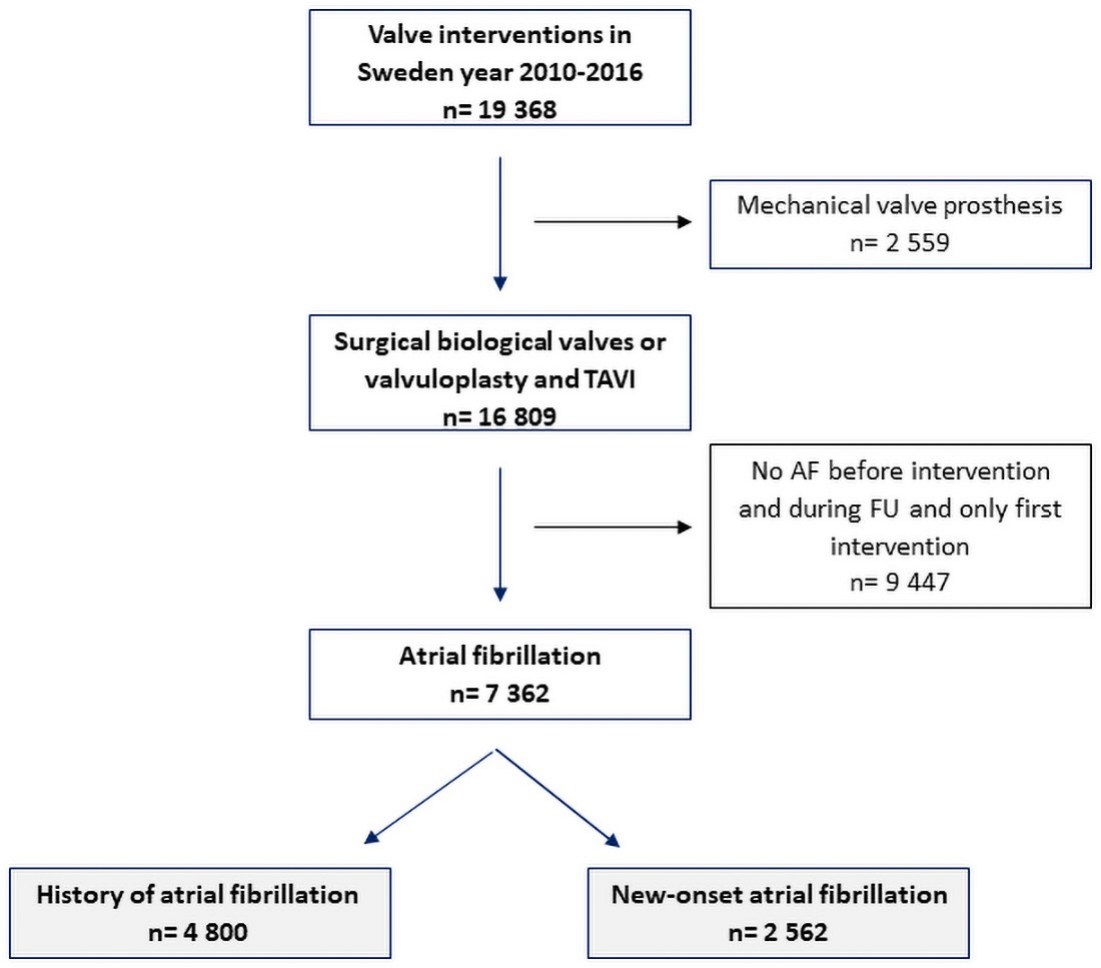
Flowchart of how the study cohort was defined.

Cardiac thoracic surgery and catheter-based valve interventions are performed at eight centers in Sweden and all patients who are residents are continuously included in the Swedish Web system for Enhancement and Development of Evidence-based care in Heart disease Evaluated According to Recommended Therapies (SWEDEHEART) registry [21]. The present study cohort included all patients in the SWEDEHEART registry undergoing surgical valve intervention with a biological prosthesis or valvuloplasty, with or without coronary artery bypass grafting (CABG), or a transcatheter valve intervention between 1 January 2010 and 31 December 2016, and who were alive at discharge after the index intervention. Patients treated with a mechanical valve prosthesis (n = 2,559) were excluded. Cardiac surgery with a biological prosthesis or a valvuloplasty was performed in 14,334 patients and 2,475 had been treated with a transcatheter valve intervention. History of AF was defined as a previous diagnosis of AF prior to the intervention or diagnosis of AF prior to discharge from the intervention (n = 4,800). New-onset AF was defined as a new diagnosis of any AF post discharge after surgery or transcatheter valve intervention (n = 2,562). The final study cohort included a total of 7,362 patients with a biological valve intervention with either history of AF or new-onset AF (Fig 1), patients not suffering AF during the study period were not included. The study was approved by the local ethics committee (Log No. 2018/481) and is in compliance with the regulations of the Declaration of Helsinki.

### Data collection

The SWEDEHEART registry contains detailed information on the procedures and concomitant diseases. Baseline information from SWEDEHEART was enriched with information from the National Patient Register (NPR), which includes diagnosis codes of all hospital admissions in Sweden since 1987 [22]. Linkage was based on the unique 10-digit personal identification number assigned to all Swedish residents at birth or immigration. The National Board of Health and Welfare approved the merging of the registries. All patients were followed through computerized linkage between the databases and the Swedish Cause of Death Register (CDR) and NPR, all managed by the Swedish National Board of Health and Welfare. The start date was the first day after discharge from the index intervention. The study cohort was followed until death or the end of follow-up (31 December 2017), whichever occurred first.

### Atrial fibrillation cohorts

History of AF was defined as a diagnosis of AF based on ICD codes from the NPR up to 3 years before and up until time of discharge from valvular intervention. New-onset AF was defined as any AF diagnosis based on ICD codes from the NPR after discharge from valvular intervention.

### Baseline information and comorbidities

Information on baseline characteristics and previously diagnosed comorbidities were collected from the SWEDEHEART registry and was enriched with data from NPR up to 3 years before the valve intervention. Predefined comorbidities were: congestive heart failure, hypertension, diabetes mellitus, ischemic stroke, TIA, myocardial infarction, chronic kidney disease, cancer, thromboembolism (systemic embolism, pulmonary embolism and venous thromboembolism) and major bleeding (hemorrhagic stroke and hospitalization for other major bleeding event). The International Code of Disease, Tenth Revision (ICD-10) was applied to identify comorbidities (S1 Table). Information regarding type of valve intervention and kidney function (creatinine) was collected from SWEDEHEART.

### Oral antithrombotic exposure

Information on the dispensation of oral anticoagulant treatment was collected by computerized linkage with the Dispensed Drug Register, which contains information on every prescription and dispensation of drugs in all pharmacies in Sweden. OAC treatment before valve intervention was defined as a dispension within 6 months prior to admission. For all analyses and for patients with a history of AF, antithrombotic treatment at baseline was defined as the drug dispensed within 1 week after discharge from valve intervention if no antithrombotic exposure prior to intervention was recorded. For patients with new-onset AF, antithrombotic treatment at baseline was defined as the drug that was dispensed within 1 week from diagnosis of AF (if no exposure prior to diagnosis was recorded). The patient was considered exposed to the corresponding dispensed antithrombotic treatment for 120 days after each dispensation as previously described [9]. Information on dispensation was continuously updated, and patients could be exposed to different pharmaceutical treatment during follow-up. The constructed variable regarding exposure to oral antithrombotic treatment was categorized as 1) warfarin treatment, 2) NOAC treatment, including apixaban, rivaroxaban, edoxaban or dabigatran, and 3) no OAC treatment. In addition to these three categories, prescription of oral antiplatelet therapy (aspirin and/or P2Y12 inhibitors) was described using the same algorithm for exposure as above.

### Statistical methods

Demographics and other baseline characteristics were summarized using frequencies for categorical variables and median and 25th and 75th percentiles for continuous variables. For tests of differences among groups, the chi-2 test was used for categorical variables and the Kruskal–Wallis test was used for continuous variables. All statistical tests were 2-tailed and performed at the 0.05 significance level. The cumulative rate of and the median time to onset of AF, as well as the proportion of new-onset AF within three months and one year was estimated and plotted. The association between initial oral anticoagulant treatment and baseline characteristics, valve position, and valve intervention type adjusting for year of inclusion were examined using a multivariable multinomial logistic regression model using no treatment OAC as reference.

The proportion of the study time spent on warfarin, NOAC, or not at any oral anticoagulants, respectively, conditional on initial treatment group was illustrated graphically in a bar chart. R version 3.5.0 statistical software (www.r-project.org) was used for statistical analyses.

## Results

### Baseline characteristics of the AF groups at valve intervention

Baseline characteristics for the patients with history of AF and new-onset AF are described in Table 1. Patients with history of AF had a median age of 75 (68–81) years at valve intervention compared to 73 (66–79) years in the new-onset AF group (p<0.001). The proportion of patients with a medical history of heart failure was higher in patients with history of AF (37.9%) compared to those with new-onset AF (15.8%) (p<0.001). Minor differences between the AF groups were found for medical history of hypertension (53.5% vs 43.5%), previous myocardial infarction (15.7% vs 12.4%), ischemic stroke (6.8% vs 4.7%) and diabetes mellitus (16.9% vs 14.5%). A CHA_2_DS_2_-VASc ≥ 2 was found in 3,988 (83.1%) and 1,934 (75.5%) in the history of AF and new-onset AF groups, respectively (Table 1). The proportion of previous intracranial bleeding was similar between both AF groups, whereas medical history of gastrointestinal bleeding and other major bleeding was more common in patients with history of AF (Table 1).

**Table 1:** Baseline characteristics of the patients with a history and new onset atrial fibrillation.

	History of Atrial fibrillation	New-onset Atrial fibrillation	p-value*
	n = 4800	n = 2562	
Age, years (median (IQR))	75 (68–81)	73 (66–79)	<0.001
Sex; Male n (%)	3075 (64.1)	1656 (64.6)	0.62
Female n (%)	1725 (35.9)	906 (35.4)	
**Medical history**			
Congestive heart failure n (%)	1820 (37.9)	406 (15.8)	<0.001
Hypertension n (%)	2567 (53.5)	1115 (43.5)	<0.001
Diabetes mellitus n (%)	809 (16.9)	372 (14.5)	0.009
Ischemic stroke n (%)	328 (6.8)	120 (4.7)	<0.001
TIA^§^ n (%)	204 (4.2)	88 (3.4)	0.09
Myocardial infarction n (%)	753 (15.7)	317 (12.4)	<0.001
Myocardial infarction within 12 months n (%)	703 (14.6)	159 (6.2)	<0.001
Peripheral artery disease n (%)	352 (7.3)	169 (6.6)	0.24
Systemic embolism n (%)	33 (0.7)	5 (0.2)	0.005
Chronic kidney disease n (%)	325 (6.8)	124 (4.8)	<0.001
Cancer n (%)	241 (5.0)	98 (3.8)	0.020
Intracranial bleeding n (%)	61 (1.3)	33 (1.3)	0.95
Gastrointestinal bleeding n (%)	302 (6.3)	108 (4.2)	<0.001
Other major bleeding n (%)	335 (7.0)	114 (4.4)	<0.001
CHA_2_DS_2_-VASc score;			<0.001
0	254 (5.3)	221 (8.6)	
1	558 (11.6)	407 (15.9)	
2	778 (16.2)	612 (23.9)	
≥3	3210 (66.9)	1322 (51.6)	
HAS-BLED score;			<0.001
0–2	3953 (82.4)	2230 (87.0)	
≥3	847 (17.6)	332 (13.0)	

*P-value by Wilcoxon or Pearson´s chi^2^ tests.

^§^TIA; transient ischemic attack.

### Valve position and intervention type in the AF groups

Tricuspid and mitral valve interventions were less frequent in the new-onset AF group compared to the history of AF group (Table 2). In total, 1,870 (73.0%) patients with new-onset AF had undergone surgical biological valve prosthesis compared to 2,605 (54.3%) among patients with a history of AF (p<0.001). (Table 2).

**Table 2:** Valve position and intervention type in the previous history and new-onset atrial fibrillation groups.

	History of Atrial fibrillation	New-onset Atrial fibrillation	p-value*
	n = 4800	n = 2562	
**Valve position** n (%)			
Aortic	3434 (71.5)	1993 (77.8)	<0.001
Mitral	1397 (29.1)	626 (24.4)	<0.001
Tricuspid	595 (12.4)	137 (5.3)	<0.001
Pulmonalis	24 (0.5)	6 (0.2)	0.09
**Intervention type** n (%)			
Surgical biological valve prosthesis	2605 (54.3)	1870 (73.0)	<0.001
Surgical valvuloplasty	1359 (28.3)	581 (22.7)	<0.001
Transcatheter biological valve prosthesis	1051 (21.9)	183 (7.1)	<0.001

*P-value by Wilcoxon or Pearson´s chi^2^ tests.

### Occurrence of new-onset AF after valve intervention

**Fig 2:**
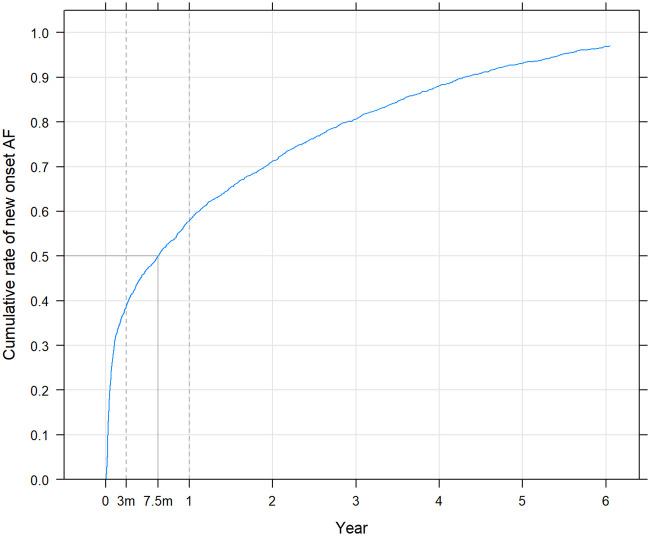
The time from discharge from valve intervention to diagnosis of new-onset atrial fibrillation. The dotted line indicate the three months period after valve intervention.

Median time from valve intervention to diagnosis of new-onset AF was 230 days. In total, 999 (39.0%) patients experienced new-onset AF within 3 months from valve intervention, and 1,484 (57.9%) patients developed new-onset AF within 1 year from intervention (Fig 2).

### Exposure to oral anticoagulant treatment after valve intervention

**Fig 3:**
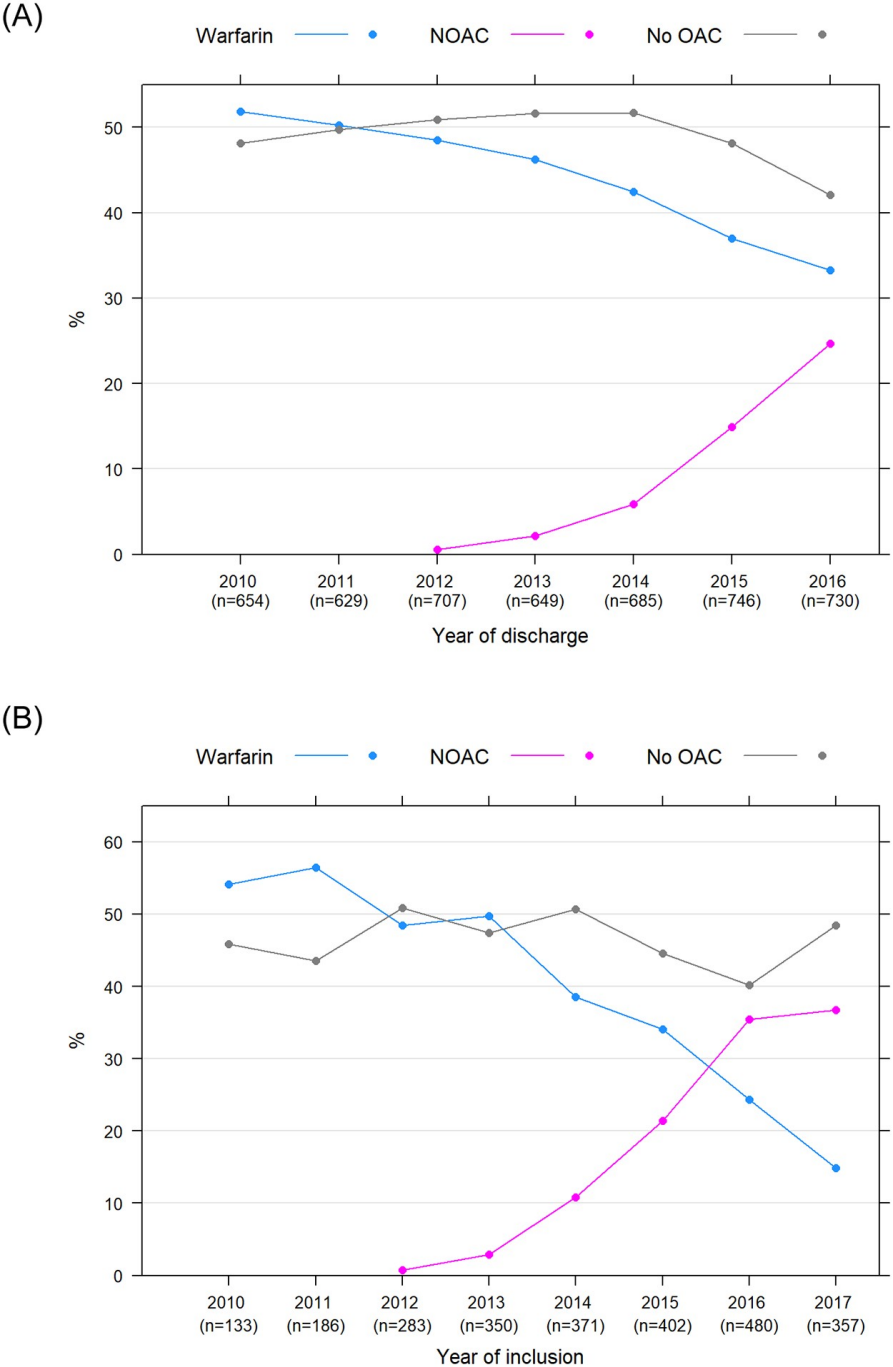
The proportion of patients with atrial fibrillation and dispensation of oral anticoagulant treatment. The proportion of patients with history of atrial fibrillation and dispensation of oral anticoagulant treatment (A) at discharge from valve intervention. The proportion of patients with new-onset atrial fibrillation and dispensation of oral anticoagulant treatment (B) at start of the atrial fibrillation. Warfarin (blue line), NOAC (purple line) and no OAC (grey line).

During the study period, 2,108 (43.9%) patients with history of AF were treated with warfarin, 349 (7.3%) were treated with NOAC, and 2,343 (48.8%) had no dispensation of OAC at discharge after valve intervention. There was a shift over time in the dispensation of warfarin and NOAC at discharge during the study period. Dispensation of warfarin decreased from 51.8% in 2010 to 33.3% in 2016, whereas dispensation of NOAC increased from 0.0% in 2010 to 24.7% in 2016 (Fig 3A).

In the group with new-onset AF, 938 (36.6%), 439 (17.1%) and 1,185 (46.3%) patients were treated with warfarin, NOAC or no OAC at diagnosis, respectively. For details of prescription of individual NOACs, see S2 Table. The dispensation pattern changed during the study period with a reduction of patients dispensed with warfarin from 54.1% in 2010 to 14.8% in 2017. Correspondingly, there was an increase in the proportion of patients dispensed with NOAC at discharge from 0.0% in 2010 to 36.7% in 2017 (Fig 3B).

A subgroup analysis in patients with a history of ischemic heart disease (myocardial infarction within 12 months, percutaneous coronary intervention and CABG) was performed and the treatment patterns with OAC were similar to the total study cohort (S3 Table).

### Clinical characteristics in relation to OAC treatment

The patients dispensed with NOAC were older and more frequently women compared to patients on warfarin (Table 3). There was no significant difference in creatinine levels when comparing the NOAC and warfarin groups (95 (79–122) vs 97 (79–126) μmol/L, p = 0.11). The proportion of patients with heart failure was lower and medical history of previous major bleeding events was more common in the NOAC group compared to the warfarin group (Table 3). The patients with no dispensation of OAC had the highest creatinine, 104 (82–142) μmol/L, and a higher proportion of previous history of chronic kidney disease, cancer, heart failure and myocardial infarction compared to the warfarin and NOAC groups (Table 3). Also, history of major bleeding was more common in the group without OAC compared to the two other OAC groups. A CHA_2_DS_2_-VASc score ≥2 was found in 2,346 (77.0%), 661 (83.9%) and 2,915 (82.6%) of the patients treated with warfarin, NOAC and no OAC, respectively. A HAS-BLED score ≥3 was observed in 417 (13.7%), 126 (16.0%) and 636 (18.0%) of the patients treated with warfarin, NOAC and no OAC, respectively (Table 3). Dispension of OAC before inclusion was more common in patients treated with warfarin (70.6%) and NOAC (65.7%) compared to no OAC (45.8%). Surgical biological valve prosthesis was more common amongst patients treated with NOAC (489 [62.1%]), compared to warfarin (1,777 [58.3%]). Valvuloplasty was more common amongst patients treated with warfarin (985 [32.3%]) compared to NOAC (142 [18.0%]) or no OAC (813 [23.0%]). Transcatheter valve intervention had been performed more often in the NOAC group (174 [22.1%]), compared to the warfarin (410 [13.5%]) and no OAC (650 [18.4%]). Clinical characteristics and dispensation of OAC in aortic and mitral valve interventions are described in the supplement (S4 and S5 Tables).

**Table 3:** Clinical characteristics and oral anticoagulant treatment.

	Warfarin	NOAC	No OAC	p-value*
	N = 3046	N = 788	N = 3528	
Age, years (median(IQR))	73 (67–79)	75 (68–80)	75 (68–81)	<0.001
Sex; Male n (%)	2071 (68.0)	483 (61.3)	2177 (61.7)	<0.001
Female n (%)	975 (32.0)	305 (38.7)	1351 (38.3)	
Creatinine (μmol/L)	97 (79–126)	95 (79–122)	104 (82–142)	<0.001
**Medical history**				
Congestive heart failure n (%)	882 (29.0)	196 (24.9)	1148 (32.5)	<0.001
Hypertension n (%)	1467 (48.2)	402 (51.0)	1813 (51.4)	0.028
Diabetes mellitus n (%)	467 (15.3)	115 (14.6)	599 (17.0)	0.097
Ischemic stroke n (%)	174 (5.7)	44 (5.6)	230 (6.5)	0.32
TIA n (%)	112 (3.7)	39 (4.9)	141 (4.0)	0.26
Myocardial infarction n (%)	407 (13.4)	107 (13.6)	556 (15.8)	0.016
Myocardial infarction within 12 months n (%)	336 (11.0)	76 (9.6)	450 (12.8)	0.016
Peripheral artery disease n (%)	180 (5.9)	60 (7.6)	281 (8.0)	0.004
Systemic embolism n (%)	23 (0.8)	2 (0.3)	13 (0.4)	0.051
Chronic kidney disease n (%)	134 (4.4)	38 (4.8)	277 (7.9)	<0.001
Cancer n (%)	118 (3.9)	28 (3.6)	193 (5.5)	0.003
Intracranial bleeding n (%)	25 (0.8)	14 (1.8)	55 (1.6)	0.012
Gastrointestinal bleeding n (%)	121 (4.0)	48 (6.1)	241 (6.8)	<0.001
Other major bleeding n (%)	139 (4.6)	33 (4.2)	277 (7.9)	<0.001
CHA_2_DS_2_-VASc score;				<0.001
0	230 (7.6)	41 (5.2)	204 (5.8)	
1	470 (15.4)	86 (10.9)	409 (11.6)	
2	569 (18.7)	166 (21.1)	655 (18.6)	
≥3	1777 (58.3)	495 (62.8)	2260 (64.1)	
HAS-BLED score;				<0.001
0–2	2629 (86.3)	664 (84.0)	2892 (82.0)	
≥3	417 (13.7)	126 (16.0)	636 (18.0)	
OAC treatment before inclusion	2149 (70.6)	518 (65.7)	1615 (45.8)	<0.001
**Intervention type** n (%)				
Surgical biological valve prosthesis	1777 (58.3)	489 (62.1)	2209 (62.6)	0.001
Surgical valvuloplasty	985 (32.3)	142 (18.0)	813 (23.0)	<0.001
Transcatheter biological valve prosthesis	410 (13.5)	174 (22.1)	650 (18.4)	<0.001

*P-value by Kruskal-Wallis or Pearson´s chi^2^ tests.

Predictors for initiating warfarin or NOAC versus no OAC are presented in Table 4. Medical history of thromboembolism and a mitral valve intervention were both predictors significantly associated with initiation of warfarin treatment versus no OAC treatment. In contrast, higher creatinine levels, female sex, and medical history of bleeding associated with no initiation of warfarin (Table 4). The type of valve intervention and a medical history of myocardial infarction did not predict warfarin treatment versus no OAC treatment. New-onset AF was the only predictor significantly associated with initiation of NOAC treatment versus no OAC treatment. In contrast, higher creatinine levels and tricuspid valve intervention associated with no initiation of NOAC (Table 4).

**Table 4:** Independent predictors of treatment with OAC versus no treatment with oral anticoagulants.

	Warfarin versus no OAC		NOAC versus no OAC	
	Odds ratio	p-value	Odds ratio	p-value
	(95% C.I.)*		(95% C.I.)*	
Age^β^	1.02 (0.96–1.08)	0.584	1.00 (0.90–1.11)	0.968
Sex, female	0.70 (0.63–0.78)	<0.001	0.89 (0.74–1.08)	0.232
Creatinine ^#^	0.71 (0.65–0.78)	<0.001	0.62 (0.53–0.73)	<0.001
**Medical history**				
Congestive heart failure	0.91 (0.81–1.02)	0.111	1.00 (0.81–1.23)	0.995
Hypertension	1.11 (0.99–1.24)	0.066	1.08 (0.89–1.30)	0.439
Diabetes mellitus	1.11 (0.96–1.28)	0.161	0.85 (0.66–1.09)	0.202
Ischemic stroke	0.89 (0.72–1.10)	0.279	1.08 (0.75–1.57)	0.679
TIA	1.02 (0.79–1.33)	0.855	1.28 (0.85–1.93)	0.238
Myocardial infarction	0.92 (0.79–1.07)	0.267	0.99 (0.76–1.27)	0.908
Peripheral artery disease	0.81 (0.66–1.00)	0.047	1.10 (0.80–1.53)	0.552
Thromboembolism	2.89 (1.43–5.85)	0.003	1.43 (0.29–6.97)	0.655
Chronic kidney disease	0.88 (0.69–1.12)	0.293	0.84 (0.56–1.25)	0.385
Cancer	0.75 (0.59–0.96)	0.023	0.80 (0.52–1.24)	0.322
Intracranial bleeding	0.53 (0.33–0.87)	0.012	1.33 (0.68–2.62)	0.406
Gastrointestinal bleeding	0.66 (0.52–0.84)	<0.001	1.00 (0.70–1.44)	0.993
Other major bleeding	0.68 (0.54–0.85)	<0.001	0.72 (0.48–1.10)	0.126
**Valve position**				
Aortic	0.75 (0.56–0.99)	0.045	1.11 (0.61–2.02)	0.738
Mitral	1.32 (1.02–1.71)	0.034	0.88 (0.51–1.50)	0.636
Tricuspid	0.78 (0.63–0.97)	0.027	0.53 (0.33–0.85)	0.009
Pulmonalis	0.31 (0.12–0.82)	0.018	0.40 (0.05–3.31)	0.394
**Intervention type**				
Surgical biological valve prosthesis	1.03 (0.74–1.43)	0.855	0.87 (0.43–1.78)	0.706
Surgical valvuloplasty	1.08 (0.77–1.51)	0.673	1.00 (0.50–2.02)	0.992
Transcatheter biological valve prosthesis	0.91 (0.63–1.32)	0.612	0.86 (0.40–1.84)	0.690
New-onset atrial fibrillation	0.96 (0.85–1.07)	0.451	1.60 (1.32–1.95)	<0.001

*Odds ratios and p-value for multinominal logistic regression for anticoagulant treatment the model including baseline characteristics, valve position, and valve intervention type, adjusting for year of inclusion. No OAC was used as reference.

^β^for a 10 year increase.

^#^ Creatinine (log2) upon admission for valve intervention.

### Change in oral anticoagulant treatment during follow-up

**Fig 4:**
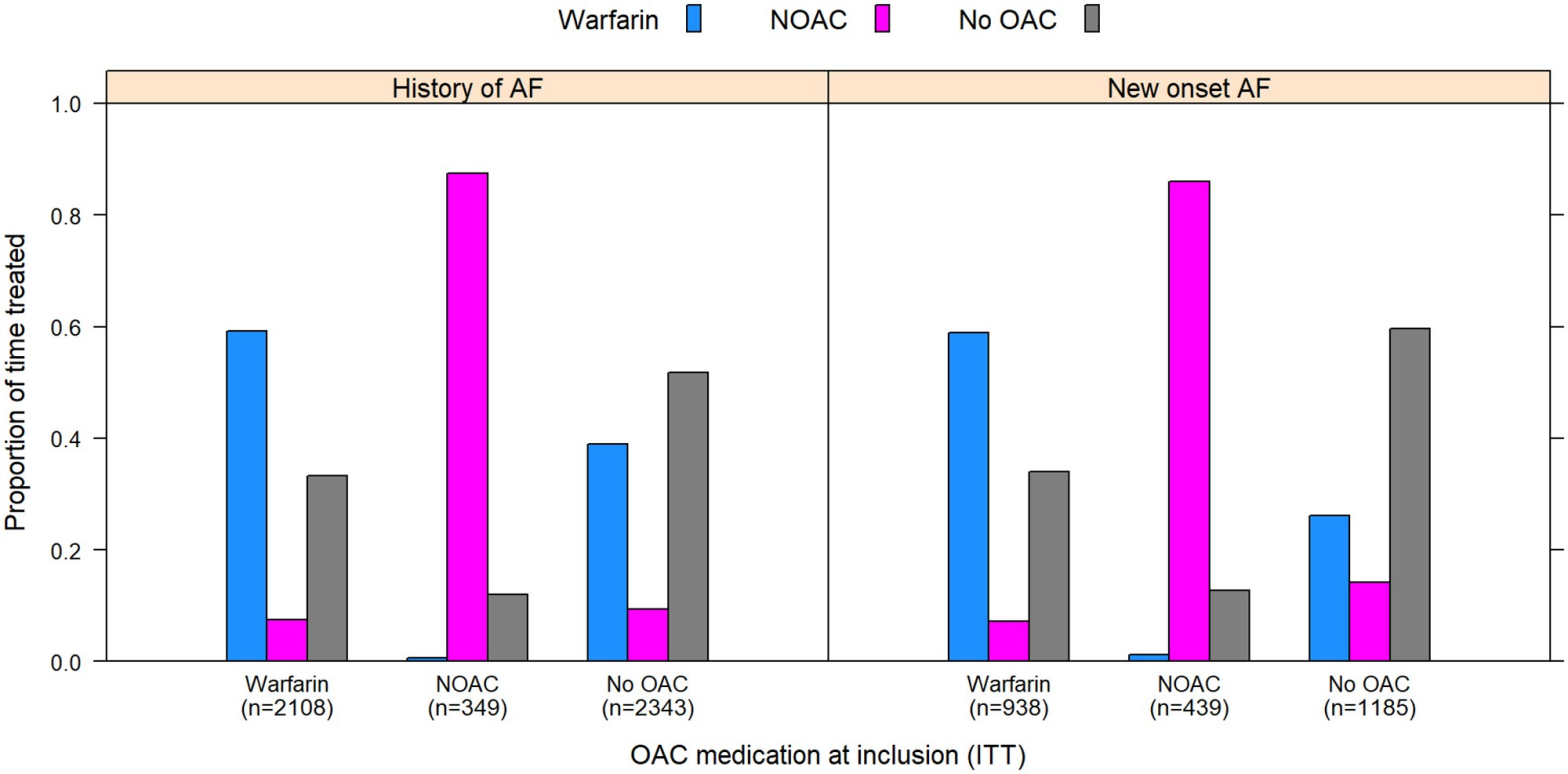
The percent of time during follow up with dispensation of oral anticoagulant treatment. Warfarin (blue), NOAC (purple) and no OAC (grey) in patients with history of atrial fibrillation separated by the OAC dispensation at discharge from valve intervention, and in patients with new-onset atrial fibrillation separated by the OAC dispensation at diagnosis of atrial fibrillation.

Exposure to oral anticoagulant treatment during follow-up (median follow-up time of 3.1 (1.7–5.1) years) was assessed for all patients in relation to anticoagulant treatment at discharge. In patients with history of AF treated with warfarin at discharge, exposure to warfarin was 59.3% of the follow-up time (Fig 4). Patients with history of AF treated with NOAC at discharge had a higher exposure to NOAC with treatment during 87.4% of the follow-up time. In the group without OAC at discharge, warfarin and NOAC were dispensed later during 38.9% and 9.4% of the follow-up time, respectively. Similar patterns were seen for patients with new-onset AF. (Fig 4).

### On-treatment patterns during follow-up

**Fig 5:**
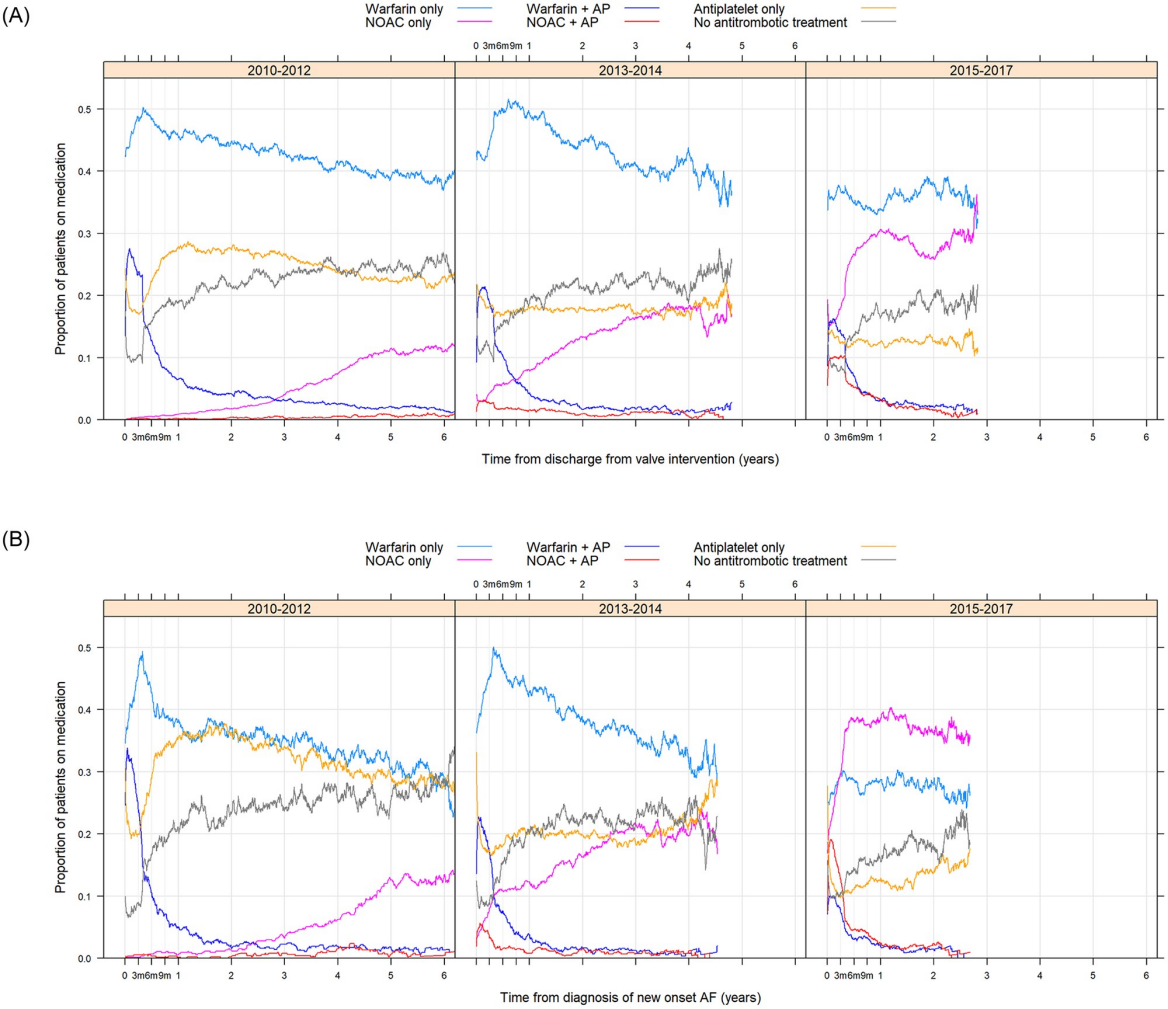
The proportion of antithrombotic exposure in the history of atrial fibrillation (A) patients exposed to warfarin, warfarin + anti-platelet (AP), NOAC, NOAC + AP, anti-platelet, no treatment during the follow-up period after discharge from valve intervention. The figure describe patients included year 2010–2012, 2013–2014 and 2015–2017. The proportion of antithrombotic exposure in the new-onset atrial fibrillation (B) patients exposed to warfarin, warfarin + AP, NOAC, NOAC + AP, anti-platelet, no treatment during the follow-up period after AF diagnosis. The figure describes the patients included year 2010–2012, 2013–2014 and 2015–2017.

Exposure to OAC was continuously updated during the study follow-up to describe antithrombotic treatment strategies after discharge post valvular intervention or after diagnosis of new-onset AF. The antithrombotic exposure was separated into warfarin, NOAC and no OAC, and also exposure to antiplatelet drugs. Since there was an increase in the general dispensation of NOAC during the study period, the analyses were divided in three time periods, year 2010–2012, 2013–2014 and 2015–2017. In patients with history of AF, the proportion exposed to NOAC was higher after the first year from valve intervention compared to the proportion at discharge. In addition, during the most recent time period 2015–2017 the increase of exposure to NOAC started 3 months post valve intervention (Fig 5A). The proportion of exposure to warfarin combined with antiplatelet drugs during the first time period in patients after valve intervention declined gradually comparing the three time periods in the study. The proportion exposed to only antiplatelet therapy or no antithrombotic treatment one year after valve intervention was around 50, 40 and 30%, respectively, in the three time periods 2010–2012, 2013–2014 and 2015–2017, with only minor changes during the total follow-up (Fig 5A). In a subgroup analysis, similar pattern was observed for patients with a medical history of ischemic heart disease (myocardial infarction within 12 months, percutaneous coronary intervention and CABG) (S1 Fig). A similar pattern for exposure of antithrombotic drugs were found in the new-onset AF group (Fig 5B).

### Outcome events during study follow-up

In the total cohort 1,754 patients died, 417 ischemic stroke, 272 myocardial infarction, 223 intracranial bleeding, 488 gastrointestinal bleedings, and 659 other major bleedings occurred during follow-up. The event rates for patients with history of AF and new-onset AF are described in Table 5.

**Table 5:** The total number and event rate of outcome events during follow up.

	History of Atrial fibrillation	New-onset Atrial fibrillation
	n (n/100 person years)	n (n/100 person years)
All-cause death	1164 (6.5)	590 (8.3)
Ischemic stroke	297 (1.7)	120 (1.7)
TIA	187 (1.0)	89 (1.2)
Myocardial infarction	169 (0.9)	103 (1.4)
Intracranial bleeding	154 (0.9)	69 (1.0)
Gastrointestinal bleeding	374 (2.1)	114 (1.6)
Urogenital bleeding	137 (0.8)	56 (0.8)
Other major bleeding	343 (1.9)	123 (1.7)

Patients treated with warfarin had an event rate of 5.2, 1.3 and 0.7 for all-cause death, ischemic stroke and myocardial infarction per 100 person years, respectively. The corresponding numbers for NOAC were 7.9, 1.6 and 1.0 per 100 person years. In contrast, patients with no OAC treatment had an event rate of 8.6 for death, 1.6 for ischemic stroke and 1.1 for myocardial infarction per 100 person years. The event rates for intracranial and gastrointestinal bleeding were 0.9 and 1.5 for patients treated with warfarin, and 0.6 and 1.7 for NOAC per 100 person years. In contrast, the event rate was 0.7 for intracranial bleeding, and 1.5 for gastrointestinal bleeding per 100 person years, for patients not receiving any treatment with OAC.

## Discussion

In this nationwide study of all patients undergoing biological valve intervention, during 2010–2016, we observed that history of AF is common but also that many patients develop new-onset AF after discharge. Importantly, we identified that a substantial part of these patients did not receive guideline recommended OAC, even though 80% of the patients had a CHA_2_DS_2_-VASc score ≥2. Higher creatinine levels and female sex were predictors of no OAC treatment. Most of the patients who were dispensed OAC received warfarin, with a shift towards a greater proportion receiving NOAC at the end of the study period.

VHD is associated with increased risk of AF. Aortic stenosis and AF share similar risk factors and might impair the physiology of the heart leading to heart failure symptoms even in patients with aortic stenosis and preserved ejection fraction [23]. The occurrence of AF in aortic stenosis is associated with worse outcomes both after surgical and transcatheter valve intervention, whereas AF is less frequently associated with poor outcome after mitral valve intervention [24–26]. In the present study, aortic stenosis accounted for the majority of the valve interventions in the open-heart surgery and in the TAVI groups. A majority of the TAVI patients had a history of AF at discharge. This is in accordance with previous studies where the TAVI cohort often has been considered a high risk group, with AF in as much as half of the patients [27]. In contrast, patients discharged without AF after surgical aortic valve intervention, had an increased risk of new-onset AF and a large proportion was diagnosed with AF during the first year. These findings emphasize the importance of repeated rhythm evaluation and monitoring during follow-up to identify patients in need for revised antithrombotic therapy and rhythm control [7].

The evidence behind the recommendations on antithrombotic strategies in AF and biological valve prostheses is limited since patients with recent surgical valve intervention were excluded from the original NOAC trials [13–16]. A recent trial comparing NOAC vs warfarin after biological mitral valve prosthesis in AF patients showed that NOAC was non-inferior to warfarin [28]. However, the majority of the patients were included in the trial beyond the first three months after surgery, a period in which NOAC still should be prescribed with caution [7, 29]. In contrast to surgical valve interventions, several studies will apply the growing evidence regarding antithrombotic treatment in TAVI where warfarin and NOAC are compared head-to-head in AF patients and initiated upon intervention [30, 31]. So far, NOAC has been found non-inferior compared to warfarin and associated with higher risk of gastrointestinal bleeding [32]. The advantage of NOAC in non-valvular AF have not yet been repeated in AF patients with recent valvular intervention. During the study period from 2010 to 2016, the majority of the AF patients were treated with warfarin as compared to NOAC. However, since 2014, the proportion of patients treated with NOAC has gradually increased both in the history of AF and new-onset AF groups. These observations are corroborated by the same trend found in other AF registries [33, 34]. The first NOAC was approved in December 2011 in Sweden, and the transition from warfarin to NOAC has overall been slow but is more pronounced in the new-onset AF group where the proportion of NOAC after 2015 is greater than warfarin. This may indicate improved adherence to updated AF guideline recommendations and that the clinicians seem to prefer NOAC when the AF occurs after the first vulnerable time period post valvular intervention [7, 35]. In patients with history of AF, the majority of clinicians still tend to prescribe warfarin over NOAC at discharge from valve intervention. However, this might change with the upcoming new TAVI trials on OAC and the ongoing shift towards choosing TAVI over open surgery in larger patient groups [30, 31]. Unfortunately, there are no ongoing antithrombotic trials in patients undergoing surgical valve interventions which limits our knowledge regarding the optimal antithrombotic treatment strategy in the early phase after surgery. Warfarin is still the preferred antithrombotic drug during this period [4].

In epidemiological studies, there is an under-prescription of OAC for stroke prevention in AF patients [36, 37]. In this valvular disease cohort with AF, where more than 80% had a CHA_2_DS_2_-VASc ≥2, as much as half of the patients with history of AF were not dispensed OAC. Previous major bleedings, female sex and higher creatinine levels were predictors of no OAC treatment. A medical history of thromboembolism and intervention of the mitral valve were predictors of warfarin treatment instead of no OAC whereas the other valve positions were not. Among the new-onset AF patients, the proportion without OAC was somewhat lower but still high and without a clear reduction over time during the study period.

Early after valve intervention there was a clear change over time in initiating antithrombotic treatment strategy where patients receiving no OAC or antiplatelet drugs were gradually fewer. However, after six months the exposure to OAC decreased and persisted at a similar level during the follow-up period. The underlying reasons for these findings need to be further examined. OAC treatment before valve intervention, patient related comorbidities indicating high bleeding risk, unawareness of AF and patients not adhering to medical treatment prescriptions are all factors that could contribute to the results.

The initial dispensation of OAC during the study period in both AF groups was the drug that patients adhered to during the majority of the study time. Patients that started treatment with warfarin only changed to NOAC during a fraction of the time. For patients initially on NOAC, the shift to warfarin was even smaller. Patients dispensed with NOAC compared to warfarin had a smaller proportion of time without OAC, which are in agreement with previous studies in AF [38]. These exposure patterns emphasize the importance of continuous cardiovascular monitoring and also patient education to improve prescription and adherence to OAC treatment [39, 40].

### Limitations and strengths

This study is based on data from national registries and the identification of the study cohort in SWEDEHEART registry may imply some limitations about data quality. However, the SWEDEHEART registry has 100% complete coverage of unselected enrollment of patients undergoing surgical valve interventions and TAVI. The registry is subject to yearly random on-site monitoring and validation and has been shown to produce high levels of agreement between the registry and available source data in the electronic health records [21]. A major limitation of this study is that the AF diagnosis is based on ICD-10 codes and not ECG registrations, as such were not available within the database. Also, it would have been interesting to define the group of patients suffering from paroxysmal postoperative AF but unfortunately this information is not available. Based on registry data, we cannot be certain that AF episodes did not occur prior to surgery in patients with a diagnosis of AF at discharge. Therefore, we have in this study not separated history of AF prior to surgery and postoperative AF during the hospital stay. Also, we do not have information on the type of AF, if Maze or left appendage occlusion procedures were performed during the surgery or the strategies chosen for AF treatment rate vs. rhythm control. However, the type of AF should not affect decision of whether or not to prescribe OAC, and limited data exists on the optimal antithrombotic strategy after left appendage occlusion [3]. The centers have their own recommendations of antithrombotic treatment for the first period after surgical valvular intervention, which might influence the prescription of OAC. The definition of drug exposure after the last dispensation of the antithrombotic drug was used as a proxy for drug intake. Pill count or actual intake cannot be guaranteed [41]. However, pharmaceuticals are involved in a comprehensive reimbursement program in Sweden, and usage of pharmaceuticals outside this program can be expected to be marginal. The reasons for change of antithrombotic treatment during the study period are not available. Finally, our study has to be interpreted with caution because of inherent limitations to all observational studies related to possible confounding by indication.

In conclusion, our study on patients discharged after valve intervention, reveals that a substantial proportion of patients have their first known episode of AF after discharge. However, irrespective of when in time AF is diagnosed, a significant proportion of patients were left untreated for stroke prevention. Warfarin was overall most frequently dispensed, especially in the first time period after discharge, but there was a shift towards using more NOAC during recent years. These findings emphasize the importance of improving evidence for choosing OAC strategy at discharge and strategies to define patients at risk of AF after valve intervention.

## Supporting information

S1 Table The International Code of Disease, Tenth Revision (ICD-10) applied to identify comorbidities. (DOCX)

S2 Table Description of the number of patients with dispension of the different NOAC at discharge from the valve intervention for the history of atrial fibrillation and upon diagnosis for the new-onset atrial fibrillation. (DOCX)

S3 Table Description of the oral anticoagulant treatment in patients with a medical history of ischemic heart disease before inclusion. (DOCX)

S4 Table Aortic valve intervention. Clinical characteristics and oral anticoagulant treatment.
(DOCX)

S5 Table Mitral valve intervention. Clinical characteristics and oral anticoagulant treatment.
(DOCX)

S1 Fig The proportion of antithrombotic exposure in the history of atrial fibrillation patients with a medical history of ischemic heart disease. Exposed to warfarin, warfarin + anti-platelet (AP), NOAC, NOAC + AP, anti-platelet, no treatment during the follow-up period after discharge from valve intervention. The figure describe patients included year 2010–2012, 2013–2014 and 2015–2017.
(DOCX)
