# Supplementary material for: Oral anticoagulant treatment after bioprosthetic valvular intervention or valvuloplasty in patients with atrial fibrillation—A SWEDEHEART study
Source: PLoS One. 2022 Jan 13;17(1):e0262580. doi: 10.1371/journal.pone.0262580 (PMC8757947; doi:10.1371/journal.pone.0262580)
Supplement: S1 Table — (DOCX) [file pone.0262580.s001.docx]

**S1 Table** The International Code of Disease, Tenth Revision (ICD-10) applied to identify comorbidities

| **Comorbidities** | **ICD-10** |
| --- | --- |
| Diabetes | E10, E11, E12, E13, E14 |
| Hypertension | I10, I11, I12, I13, I15 |
| Previous heart failure | I42, I50, I110, I255, I130, I132, K761 |
| Previous atrial fibrillation | I48 |
| Previous ischemic stroke | I63 |
| Previous TIA | G45 |
| Previous systemic embolism | I74 |
| Previous venous thromboembolism | I801, I802, I82, I26, O882, O888 |
| Previous major bleeding | Intracranial bleeding: I60, I61, I62, S064, S065, S066. Gastrointestinal bleeding: K226, K250, K252, K254, K256, K260, K262, K264, K266, K270, K272, K274, K276, K280, K282, K284, K286, K290, K625, K661, K920, K921, K922, I850, I983.  Urogenital bleeding: N02, R319, N939, N950, N501A.  Other bleeding: H113, H313, H356, H431, H450, H922, I312, J942, M250, R04, R58, T810, D500, D629. |
| Previous myocardial infarction | I21, I22, I252 |
| Previous peripheral vascular disease | I70, I71, I72, I73 |
| Previous Chronic kidney disease | N18, N17, N19, Z992 |
| Cancer | C |
| CHADS2-VA2Sc score | 1 point each for: heart failure, hypertension, age 65-74 years, diabetes, vascular disease, female sex 2 points each for 75 years, thromboembolism. |
| HAS-BLED score (modified) | 1 point each for hypertension, renal failure, liver disease, thromboembolism, any bleeding, age  ≥65 years, prescription of antiplatelet agent or NSAID, alcohol abuse. |
