# Supplementary material for: Oral anticoagulant treatment after bioprosthetic valvular intervention or valvuloplasty in patients with atrial fibrillation—A SWEDEHEART study
Source: PLoS One. 2022 Jan 13;17(1):e0262580. doi: 10.1371/journal.pone.0262580 (PMC8757947; doi:10.1371/journal.pone.0262580)
Supplement: S2 Table — (DOCX) [file pone.0262580.s002.docx]

**S2 Table** Description of the number of patients with dispension of the different NOAC at discharge from the valve intervention for the history of atrial fibrillation and upon diagnosis for the new-onset atrial fibrillation

| **NOAC** | **Total** | **History of Atrial fibrillation** | **New-onset Atrial fibrillation** |
| --- | --- | --- | --- |
| Apixaban | 551 | 239 | 312 |
| Rivaroxaban | 127 | 61 | 66 |
| Edoxaban | 2 | 0 | 2 |
| Dabigatran | 108 | 49 | 59 |
