# Supplementary material for: Oral anticoagulant treatment after bioprosthetic valvular intervention or valvuloplasty in patients with atrial fibrillation—A SWEDEHEART study
Source: PLoS One. 2022 Jan 13;17(1):e0262580. doi: 10.1371/journal.pone.0262580 (PMC8757947; doi:10.1371/journal.pone.0262580)
Supplement: S3 Table — (DOCX) [file pone.0262580.s003.docx]

**S3 Table Description of the oral anticoagulant treatment in the patients with a medical history of ischemic heart disease**

|  | **History of Atrial fibrillation**  **n (%)** | **New-onset Atrial fibrillation n (%)** |
| --- | --- | --- |
| Warfarin | 280 (39.8) | 56 (35.2) |
| NOAC | 53 (7.5) | 23 (14.5) |
| No OAC | 370 (52.6) | 80 (50.3) |
