# Supplementary material for: Oral anticoagulant treatment after bioprosthetic valvular intervention or valvuloplasty in patients with atrial fibrillation—A SWEDEHEART study
Source: PLoS One. 2022 Jan 13;17(1):e0262580. doi: 10.1371/journal.pone.0262580 (PMC8757947; doi:10.1371/journal.pone.0262580)
Supplement: S4 Table — Clinical characteristics and oral anticoagulant treatment. (DOCX) [file pone.0262580.s004.docx]

**S4 Table Aortic valve intervention** Clinical characteristics and oral anticoagulant treatment

|  | Warfarin  N=2046 | NOAC  N=654 | No OAC  N=2727 | p-value* |
| --- | --- | --- | --- | --- |
| Age, years | 76 (70-81) | 76 (70-82) | 76 (70-82) | 0.55 |
| Sex; Male n (%)  Female n (%) | 1340 (65.5)  706 (34.5) | 386 (59.0)  268 (41.0) | 1672 (61.3)  1055 (38.7) | 0.002 |
| Creatinine (µmol/L) | 99 (80-130) | 95 (79-123) | 102 (81-138) | <0.001 |
| **Medical history** |  |  |  |  |
| Congestive heart failure n (%) | 572 (28.0) | 158 (24.2) | 832 (30.5) | 0.003 |
| Hypertension n (%) | 1104 (54.0) | 354 (54.1) | 1510 (55.4) | 0.60 |
| Diabetes mellitus n (%) | 406 (19.8) | 107 (16.4) | 526 (19.3) | 0.14 |
| Ischemic stroke n (%) | 146 (7.1) | 33 (5.0) | 181 (6.6) | 0.17 |
| TIA n (%) | 87 (4.3) | 34 (5.2) | 119 (4.4) | 0.58 |
| Myocardial infarction n (%) | 322 (15.7) | 96 (14.7) | 487 (17.9) | 0.052 |
| Peripheral artery disease n (%) | 151 (7.4) | 54 (8.3) | 256 (9.4) | 0.047 |
| Systemic embolism n (%) | 21 (1.0) | 0 (0.0) | 12 (0.4) | 0.004 |
| Chronic kidney disease n (%) | 113 (5.5) | 36 (5.5) | 234 (8.2) | <0.001 |
| Cancer n (%) | 89 (4.3) | 27 (4.1) | 150 (5.5) | 0.12 |
| Intracranial bleeding n (%) | 17 (0.8) | 12 (1.8) | 34 (1.2) | 0.095 |
| Gastrointestinal bleeding n (%) | 91 (4.4) | 45 (6.9) | 197 (7.2) | <0.001 |
| Other major bleeding n (%) | 96 (4.7) | 29 (4.4) | 204 (7.5) | <0.001 |
| CHA_2_DS_2_-VASc score;  0  1  2  ≥3 | 57 (2.8)  226 (11.0)  362 (17.7)  1401 (68.5) | 23 (3.5)  60 (9.2)  128 (19.6)  443 (67.7) | 111 (4.1)  265 (9.7)  485 (17.8)  1866 (68.4) | 0.16 |
| HAS-BLED score;  0-2  ≥3 | 1702 (83.2)  344 (16.8) | 541 (82.7)  113 (17.3) | 2207 (80.9)  520 (19.1) | 0.12 |

*P-value by Kruskal-Wallis or Pearson´s chi^2^ tests.
