# Supplementary material for: Oral anticoagulant treatment after bioprosthetic valvular intervention or valvuloplasty in patients with atrial fibrillation—A SWEDEHEART study
Source: PLoS One. 2022 Jan 13;17(1):e0262580. doi: 10.1371/journal.pone.0262580 (PMC8757947; doi:10.1371/journal.pone.0262580)
Supplement: S5 Table — Clinical characteristics and oral anticoagulant treatment. (DOCX) [file pone.0262580.s005.docx]

**S5 Table Mitral valve intervention** Clinical characteristics and oral anticoagulant treatment

|  | Warfarin  N=1048 | NOAC  N=148 | No OAC  N=827 | p-value* |
| --- | --- | --- | --- | --- |
| Age, years | 68 (62-74) | 69 (63-73) | 71 (64-76) | <0.001 |
| Sex; Male n (%)  Female n (%) | 776 (74.0)  272 (26.0) | 106 (71.6)  42 (28.4) | 535 (64.7)  292 (35.3) | <0.001 |
| Creatinine (µmol/L) | 95 (78-122) | 93 (77-122) | 109 (85-160) | <0.001 |
| **Medical history** |  |  |  |  |
| Congestive heart failure n (%) | 321 (30.6) | 42 (28.4) | 324 (39.2) | <0.001 |
| Hypertension n (%) | 390 (37.2) | 53 (35.8) | 327 (39.5) | 0.50 |
| Diabetes mellitus n (%) | 65 (6.2) | 11 (7.4) | 84 (10.2) | 0.007 |
| Ischemic stroke n (%) | 31 (3.0) | 11 (7.4) | 57 (6.9) | <0.001 |
| TIA n (%) | 28 (2.7) | 7 (4.7) | 22 (2.7) | 0.34 |
| Myocardial infarction n (%) | 100 (9.5) | 14 (9.5) | 79 (9.6) | 1.00 |
| Peripheral artery disease n (%) | 45 (4.3) | 7 (4.7) | 41 (5.0) | 0.79 |
| Systemic embolism n (%) | 4 (0.4) | 2 (1.4) | 2 (0.2) | 0.14 |
| Chronic kidney disease n (%) | 26 (2.5) | 3 (2.0) | 51 (6.2) | <0.001 |
| Cancer n (%) | 35 (3.3) | 2 (1.4) | 44 (5.3) | 0.022 |
| Intracranial bleeding n (%) | 8 (0.8) | 3 (2.0) | 22 (2.7) | 0.005 |
| Gastrointestinal bleeding n (%) | 32 (3.1) | 5 (3.4) | 50 (6.0) | 0.006 |
| Other major bleeding n (%) | 46 (4.4) | 4 (2.7) | 70 (8.5) | <0.001 |
| CHA_2_DS_2_-VASc score;  0  1  2  ≥3 | 170 (16.2)  244 (23.3)  214 (20.4)  420 (40.1) | 21 (14.2)  25 (16.9)  42 (28.4)  60 (40.5) | 86 (10.4)  138 (16.7)  180 (21.8)  423 (51.1) | <0.001 |
| HAS-BLED score;  0-2  ≥3 | 962 (91.8)  86 (8.2) | 131 (88.5)  17 (11.5) | 698 (84.4)  129 (15.6) | <0.001 |

*P-value by Kruskal-Wallis or Pearson´s chi^2^ tests.
