# Supplementary material for: Oral anticoagulant treatment after bioprosthetic valvular intervention or valvuloplasty in patients with atrial fibrillation—A SWEDEHEART study
Source: PLoS One. 2022 Jan 13;17(1):e0262580. doi: 10.1371/journal.pone.0262580 (PMC8757947; doi:10.1371/journal.pone.0262580)

**S1 Fig The proportion of antithrombotic exposure in the history of atrial fibrillation** **patients with a medical history of ischemic heart disease**


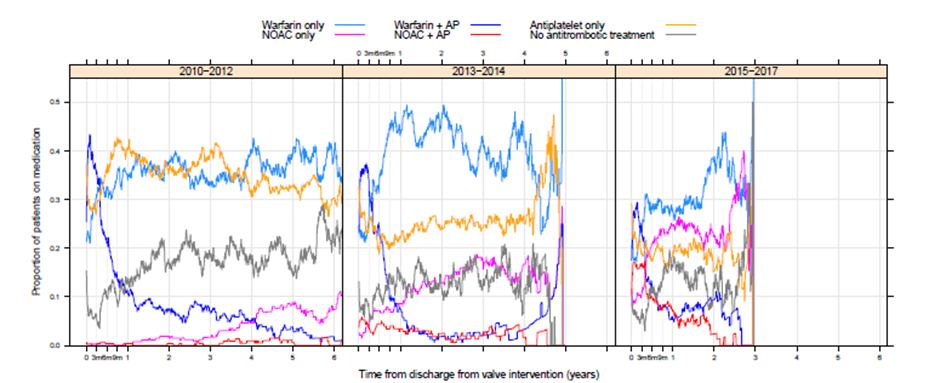

Supplement: S1 Fig — Exposed to warfarin, warfarin + anti-platelet (AP), NOAC, NOAC + AP, anti-platelet, no treatment during the follow-up period after discharge from valve intervention. The figure describe patients included year 2010–2012, 2013–2014 and 2015–2017. (DOCX) [file pone.0262580.s006.docx]
